# No Mendelian Genes in Psychiatry?

**DOI:** 10.20900/jpbs.20210019

**Published:** 2021-10-27

**Authors:** José V. Pardo

**Affiliations:** 1 Cognitive Neuroimaging Unit, Mental Health Service Line, Minneapolis VA Health Care System, Minneapolis, MN 55417, USA; 2 Department of Psychiatry, University of Minnesota School of Medicine, Minneapolis, MN 55455, USA

**Keywords:** monogenic, polygenic, Mendelian inheritance, genome wide association studies (GWAS), copy number variant (CNV), consanguinity, homozygosity mapping, VMAT2, depression, monoamine hypothesis

## Abstract

To date, no gene following Mendelian inheritance (e.g., monogenic variant) has been discovered for any common psychiatric disorder. This unfortunate circumstance has delayed meaningful inroads into the pathophysiology of psychiatric disease that has otherwise enabled advances into so many other fields of medicine. New methods and approaches can now find these putative genes offering the same potential for foundational impact on psychiatry as has occurred in diverse fields such as metabolism, hematology, and cancer. If unsuccessful, a significant impediment in progress toward mitigating the suffering from mental illness will result.

## INTRODUCTION

Traits or phenotypes can be controlled by a single genetic locus (monogenic) and can follow a simple inheritance pattern. In such cases, a mutation in a single gene can cause a disease inherited according to the principles identified by Mendel (i.e., Mendelian gene) [1]. Dominant diseases manifest in heterozygotes; recessive diseases in homozygotes. Mendelian genes, whether recessive or dominant (both autosomal and X-linked), are the foundation of human genetics, catalogued in Victor McKusick’s “Mendelian Inheritance in Man” OMIM [2]. Discovery of such genes, often based on astute clinical observation rather than quantitative research, was foundational in understanding disease pathophysiology and mechanisms yielding critical information for medical progress, novel therapeutics, and personalized medicine. These discoveries motivated the Human Genome Project. Where are those genes in psychiatry?

Psychiatric leadership consenses no single gene causes any mental disorder or determines any behavioral variant. The prevailing view is that mental illness is purely polygenic with hundreds or thousands of genes of small effect size. However, the tenet that Mendelian genes do not exist in psychiatry warrants caution; the claim is far from proven and awaits empirical demonstration.

The long list of risk variants for mental disorders, hundreds in number, come principally from Genome Wide Association Studies (GWAS). Given the common disease/common gene hypothesis, finding a statistically significant association of a genetic locus when comparing patients with a diagnosis of a disorder (e.g., Diagnostic and Statistical Manual, DSM) to a control group appears compelling. Inherent in this approach is the assumption a DSM diagnosis is a singular biological disease. In other words, the variability in phenotype seen in a DSM diagnosis arises most likely from variable expressivity at a locus (e.g., some patients with schizophrenia have mostly auditory hallucinations, while other have mostly negative symptoms).

Alternatively, if a DSM “diagnosis” is a term for a syndrome describing a set of phenotype(s) associated with more than one distinct biological disease (i.e., genetic heterogeneity), GWAS would also provide many risk variants with small effect sizes even if all the diseases were monogenic. For any given protein, missense risk variants in a gene can occur at any of the hundreds or thousands of amino acids coding functional domains. In this case, accurate diagnosis requires improved disease definition. For example, the empirical “diagnosis” of mild cognitive impairment (MCI) is used to describe preclinical or early disease whether biologically frontotemporal, Lewy body, or Alzheimer’s dementia.

## PSYCHIATRIC GENETICS

No one believes psychiatric diseases are all monogenic. It is not anticipated single Mendelian genes cause most common mental illness. For example, complicated bereavement or stress-associated depression are both common but have lower heritability. However, it is important to at least leave open the question about whether any mental disorder is monogenic based on several considerations:

1. Why should all other fields of medicine outside of psychiatry have Mendelian genes? Is it something about psychiatry? The higher degree of shared heritability across psychiatric disorders, in comparison to neurological disorders, suggests DSM criteria do not reflect genetics [3]. This phenomenon may have historical roots: neurological disorders have generally had more observable and distinctive features (epileptic foci, protein deposits) than those seen in mental illness. Is it something about the brain’s complexity? Unlikely, given the presence of monogenic brain disorders such as Fragile X, Rett’s, or Angelman’s syndromes.
2. DSM nosology was developed for practical clinical application. Although mostly devoid of rigorous empirical evidence, this diagnostic system at least used as a framework the Feighner Research Criteria [4]. Feighner et al.’s expert consensus criteria identified only 14 disorders compared with DSM’s more than 150 entries. DSM as applied in the research setting is increasingly questioned and may not map clinical labels onto biology. Some suggest that mental disorders are only extremes in a continuum of constructs from cognitive neuroscience (i.e., RDoc). If so, psychiatry would be unique in the medical field in not having any categorical diagnoses or diagnostic boundaries.
3. There are concerns about pure polygenic models explaining the low odds ratios from GWAS. For example, Hodge and Greenberg (2017) proposed a quantitative model assuming many disease susceptibility loci each independently contributing an equal, small, and additive amount to disease susceptibility [5]. Based on this model and assuming reasonable disease prevalence and threshold liability, the low odd-ratios observed frequently in GWAS (<1.2) would require a very large number of risk loci with high gene frequencies that they considered biologically implausible. An alternative more viable model was proposed: genetic heterogeneity.
4. The translation of polygenic etiologies for psychiatry, as in most fields, remains daunting and requires entirely new scientific approaches and “big data.”
5. The explosion in the number of Mendelian disorders makes clear single genes often affect multiple organ systems and signaling cascades [6]. Research on psychosis can model recent approaches into oncology’s molecular dissection of malignancy and impact of cell signaling to make scientific progress and to develop novel therapeutics for personalized medicine.
6. The study of consanguineous families, where current methods are most likely to find Mendelian genes, has been limited. High rates of consanguinity occur in Africa; the Middle East; and west, central, and south Asia [7]. International collaboration, despite difficulties, is essential. The frequency of consanguinity is decreasing as risks for recessive diseases become fully appreciated. Through identity by descent, consanguinity enables identification of rare autosomal recessive genes [8]. A separate issue is the target disorder or phenotype for the study of linkage. Given the rarity of such recessive genes, a mutation would essentially define the illness. If the careful study of many multiplex consanguineous families with apparent recessive transmission of psychiatric disease lacks Mendelian genes, perhaps the null hypothesis must be accepted.
7. Studies of large and small nuclear families as well as of twins have proven historically useful in the search for Mendelian genes. However, incomplete penetrance and phenotypic heterogeneity of mental disorders have complicated dissection (e.g., DISC1) and will continue to frustrate the search for Mendelian genes. In the case of monogenic pedigrees, the variance in phenotype can provide an estimate of the penetrance/phenotypic heterogeneity of the mental illness.
8. Although rare, there are already well-established examples of monogenic neuropsychiatric disorders that have prominent symptoms of mood, affect, and personality dysfunction (Huntington’s; autosomal dominant Parkinson’s and Alzheimer’s disease; Angelman’s). Gene discovery has produced highly significant impact even fertilizing other fields.
9. A major advance in psychiatric genetics is the discovery of the association of several psychiatric disorders with copy number variants (CNVs), both familial and de novo. These are typically, but not always, polygenic and associate with neurodevelopmental disorders such as autism, schizophrenia, or intellectual disability—frequently in combination [9]. CNVs may be more common in psychiatric disorders than appreciated, as it has been possible only recently to detect the most common, small, structural variants using long-read technology. There is also evidence that monogenic mutations, particularly in X-linked genes such as NLGN4, may be causal to neurodevelopmental disorders [10]. These findings suggest that even if classical Mendelian autosomal recessive mutations cannot be found in association with common psychiatric disorders, genetic research strategies other than GWAS have the potential to provide insights into the pathophysiology of mental illness.
10. A limited literature hints already at the potential existence of Mendelian genes for common psychiatric disorders/phenotypes such as depression and may pave the way for future research as outlined next.

## A MENDELIAN GENE FOR DEPRESSION?

Major depression is a common DSM diagnosis, and many pathways can lead there (e.g., reserpine, substance abuse, stroke). One theory about depression since the 1960s is the “Monoamine Theory of Depression” [11,12]. Simply stated: low levels of monoamines are causal to the depression phenotype, although a precise etiology was not identified. For example, MAOA is an enzyme that metabolizes monoamines thereby decreasing synaptic availability. In fact, inhibition of this enzyme is an effective antidepressant (MAOI). Patients with depression have greatly increased brain MAOA activity providing an explanation for low synaptic levels [13]. However, despite numerous candidate gene studies and GWAS, no MAOA gene has proven associated with depression. However, one recent report suggests a plausible novel monogenetic basis for low monoamines in depression consistent with past theories [14].

A Saudi consanguineous family presented with 3 girls and five boys with infantile Parkinson’s disease, a very rare disorder. No others had Parkinson’s. The mode of transmission of Parkinson’s was recessive. The description of the index case noted “crying lasting hours.” Five of the eight consanguineous parents suffered from clinical depression. These facts by themselves could be explained by the psychological impact of the disease.

**Figure 1.**
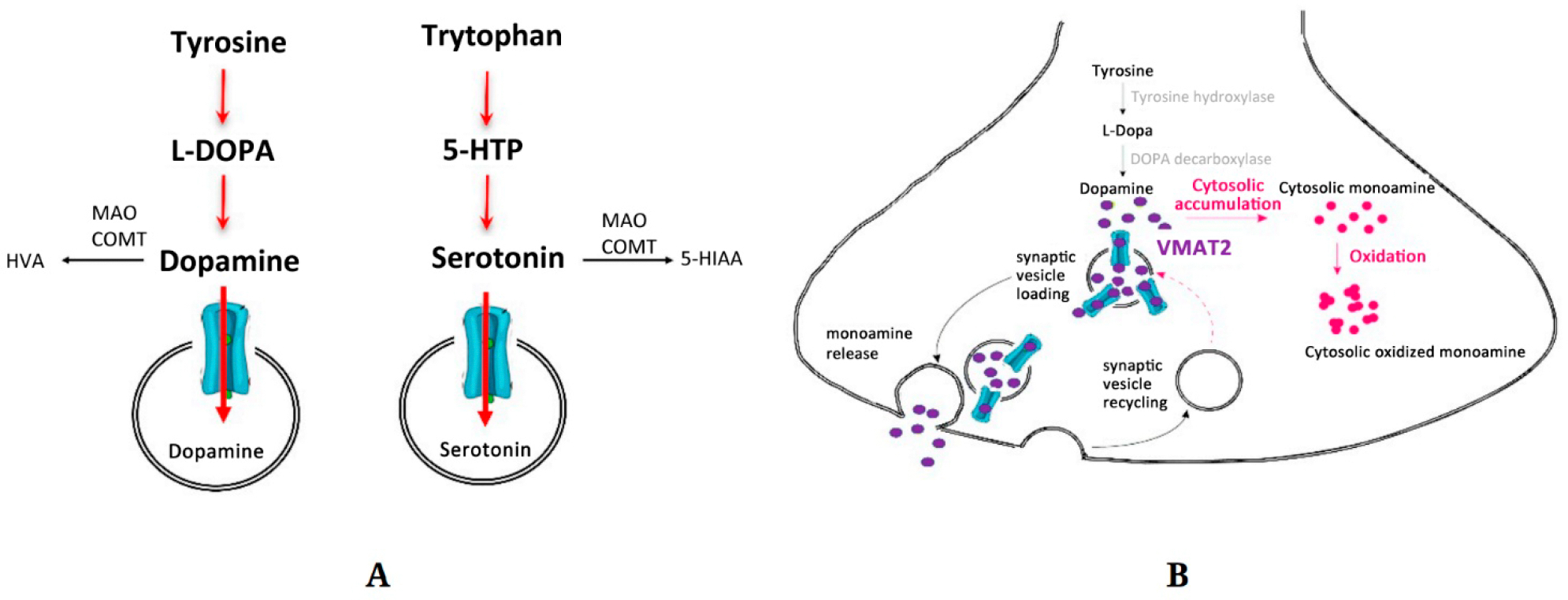
Presynaptic terminal of a monoamine neuron showing synthesis, packaging, vesicular release, and metabolism of dopamine or serotonin. (**A**) Biosynthetic and catabolic pathway. 5-HTP, 5-hydroxytryptophan; COMT, catechol-*O*-methytransferanse; HVA, homovanillic acid; 5-HIAA, 5-hydroxyindoleacetic acid; MAO, monoamine oxidase. (**B**) Cartoon displaying how VMAT2 mutation can produce decreased monoaminergic tone, Parkinsonian and depression phenotypes, and toxicity to L-DOPA. VMAT2 is the transporter moving monoamine (dopamine or serotonin) from the cytoplasm into the synaptic vesicle. The mutant VMAT2 P228L has greatly diminished transport ability and cannot load the vesicles with normal amounts of dopamine or serotonin. In turn, this causes a deficit of monoamine at the synapse. Hypodopaminergic states are associated with Parkinson’s, while hyposerotoninergic states are associated with depression. Note that treatment with L-Dopa cannot bypass the VMAT2 bottleneck. Monoamines accumulate in the cytoplasm, become oxidized, and generate toxic byproducts. Blue, VMAT2; purple, dopamine; red, oxidized dopamine/toxic aggregate byproducts. Modified from Rilstone et al. [14].

DNA was obtained; exons were sequenced; parametric linkage and homozygosity mapping produced only one pathogenic variant. The variant was not in any database of normal subjects or patients with Parkinson’s disease. The variant was homozygous in all family members with Parkinson’s but was not homozygous in 78 unaffected family members, 26 of whom carried the variant heterozygously. The mutation surfaced in the vesicular monoamine transporter, type II (VMAT2 p.Pro387Leu) that shuttles dopamine or serotonin between cytoplasm and synaptic vesicles for neurotransmission (Figure 1A). The mutant protein, engineered in vitro into vesicular expression using the techniques of molecular biology, showed greatly reduced but not absent serotonin transport. Reserpine, a drug associated with depression, could block transport even further. Possibly, the mutation is dominant with variable expressivity for the depression phenotype and recessive for Parkinson’s.

These genetic data held promise for personalized medicine. When several affected children were treated with L-dopa/carbidopa, typically used in geriatric Parkinson’s, they rapidly deteriorated. No matter how much dopamine is produced in the cytoplasm, it cannot enter the vesicle for delivery into the synapse (Figure 1B). Cytoplasmic dopamine metabolites therefore increased causing cellular toxicity. In contrast, treatment with the post-synaptic agonist, pramipexole, yielded significant and lasting improvement. Another inference of this research is that MAOI treatment would be totally ineffective and potentially toxic to the depressed parents.

This mutation is rare (MAF < (10)^−5^ gnomAD) and will not be a common cause of the depression phenotype. It does not arise in any GWAS of depression. Yet, it’s inheritance as a potential Mendelian gene provided considerable insight into pathophysiology with major implications for precision medicine. If Mendelian genes exist, as this mutation suggests, a foundational impact on psychiatric nosology, disease pathophysiology, and translation will lead to new preventive, diagnostic, and therapeutic approaches. If they do not, psychiatric genetics will be disadvantaged.

## CONCLUSIONS

The current state of psychiatric genetics posits mental illness is purely polygenic. Simple Mendelian genes associated with common psychiatric disease do not exist. However, recent advances in sequencing, use of consanguineous multiplex pedigrees with psychiatric phenotypes, and homozygosity mapping may enable in the next decade a test of the present state. As has occurred in all other fields of medicine, Mendelian genes, if discovered, will provide major foundational advances in disease pathophysiology with impact on nosology, prevention, therapeutics, and personalized medicine. If these genes do not exist, psychiatry will be challenged. Progress in psychiatric genetics must then await new methods, algorithms, and processing pipelines to identify variants of key relevance to psychiatry and approaches for translation for clinical utility.
